# Supplementation of a Multi-Carbohydrase and Phytase Complex in Diets Regardless of Nutritional Levels, Improved Nutrients Digestibility, Growth Performance, and Bone Mineralization of Growing–Finishing Pigs

**DOI:** 10.3390/ani13091557

**Published:** 2023-05-06

**Authors:** Fangyuan Chen, Lunxiang Yang, Li Zhe, Maamer Jlali, Yong Zhuo, Xuemei Jiang, Lingjie Huang, Fali Wu, Ruinan Zhang, Shengyu Xu, Yan Lin, Lianqiang Che, Bin Feng, De Wu, Aurélie Preynat, Zhengfeng Fang

**Affiliations:** 1 Key Laboratory for Animal Disease-Resistant Nutrition of the Ministry of Education of China, Chengdu 611130, China; 2 Key Laboratory of Animal Disease-Resistant Nutrition of Sichuan Province, Chengdu 611130, China; 3 Animal Nutrition Institute, Sichuan Agricultural University, Chengdu 611130, China; 4 Adisseo France SAS, Center of Expertise in Research and Nutrition, F-03600 Commentry, France

**Keywords:** multi-carbohydrase and phytase complex, nutrient digestibility, bone mineralization, growth performance, pig

## Abstract

**Simple Summary:**

Non-starch polysaccharides and phytate have been considered the most common antinutritional factors in feedstuffs, and there is no corresponding enzyme in animals. Our results showed that the multi-carbohydrase and phytase supplementation had a potent role in improving energy, amino acids, calcium, and phosphorus digestibility in fattening pigs regardless of dietary nutrient levels, thus increasing the deposition of calcium and phosphorus in bone and improving the growth rate and feed efficiency of fattening pigs. These findings have important implications for the application of multi-enzymes in fattening pigs to spare feed costs, reduce nutrient waste, and improve swine production efficiency.

**Abstract:**

This study was conducted to investigate the effects of dietary multi-enzyme (multi-carbohydrase and phytase complex, MCPC) supplementation on digestibility, growth performance, bone mineralization, and carcass yield and traits in growing–finishing pigs fed diets with adequate or deficient net energy (NE), amino acids (AA), calcium (Ca) and phosphorus (P) levels. A total of 576 crossbred [Duroc × (Landrace × Yorkshire)] barrows (~25 kg) were fed one of the six diets till live weight approached 130 kg. Basal diets included a positive control (PC), negative control 1 (NC1) and 2 (NC2), while another three diets were prepared by adding MCPC to the three basal diets. The final body weight was lower (*p* < 0.05) in NC2 than in NC1 and PC treatments, while overall feed intake and feed-gain ratio were higher (*p* < 0.05) in NC1 and NC2 than in PC treatment. The NC2 treatment showed lower (*p* < 0.05) carcass weight but higher (*p* < 0.05) lean meat percentage than the PC treatment. The apparent ileal digestibility (AID) of gross energy (GE), crude protein (CP) and AA was decreased (*p* < 0.05) or tended (*p* < 0.10) to decrease in NC1 and/or NC2 diets compared with a PC diet. MCPC supplementation improved (*p* < 0.05) AID of Ca, P and AA (Lys, Leu, Val, Phe, Gly, Tyr and Pro), apparent total-tract digestibility (ATTD) of GE, CP, bone strength, Ca, and P retention. In conclusion, MCPC supplementation improved nutrient digestibility, bone mineralization, and growth performance of fattening pigs, regardless of the nutritional level of the basal diet.

## 1. Introduction

Non-starch polysaccharides (NSP), the main components of plant cell walls, and phytate, the main form of plant-derived phosphorus (P), have long been considered the most common antinutritional factors in the major feedstuffs, including corn, soya, wheat, barley, and their by-products [1,2,3,4]. There is growing interest in using enzyme preparations to reduce the antinutritive effects of NSP and phytate, thereby increasing feed efficiency, decreasing nutrient (particularly nitrogen and P) losses via feces and urine, and minimizing the cost of dietary formulation and waste disposal [5,6,7,8,9]. It is documented that phytase can decompose phytate P into inositol and inorganic phosphorus [10,11], while carbohydrases have the potential to enhance the nutritive value of diet by alleviating the physical encapsulation of intracellular nutrients by plant cell walls and thus improving energy and amino acid availability [9]. Several studies have shown that supplementing a combination of phytase and carbohydrases in corn, wheat or barley-based diets is more beneficial with regard to P digestibility and retention than supplementation of the individual enzymes [12,13,14,15].

In China, swine nutrition is said to be a step ahead of poultry nutrition in terms of the precision of nutrient requirements in the diet. However, the use of exogenous enzymes to improve nutrient efficiency and reduce feed cost and nutrient excretion is much more common in poultry nutrition than in swine nutrition. Beyond the efficacy of individual enzymes, the supplementation of carbohydrase and phytase complex (multi-enzyme) in diets fed to poultry species has been shown to allow a much higher reduction in levels of energy and nutrients without adverse effects on the performance of the birds [16,17]. To date, only limited information is available on the efficacy of multi-enzyme in swine diets differing in nutrient levels [6]. Moreover, it remains unclear how multi-enzyme affects growth performance, bone mineralization and strength, and carcass yield and quality in growing–finishing pigs.

Corn, soybean meal, wheat, and their by-products contain much NSP and phytate. Therefore, the objective of this study was to test whether the supplementation of a multi-carbohydrase and phytase in corn-soybean meal-wheat and their by-products diets deficient in net energy, digestible amino acids, digestible P, and Ca could improve nutrient digestibility, energy availability. This could alleviate the potential adverse effects of the deficiency of energy, amino acid, Ca, and P on growth performance, bone mineralization and strength and carcass yield and quality of growing–finishing pigs.

## 2. Materials and Methods

### 2.1. Experimental Design

Before the start of the experiment, crossbred [Duroc × (Landrace × Yorkshire)] piglets (castrated males) were selected from a single weaning batch and fed with the same diets (Supplemental Table S1) until the mean body weight of pigs approached 25 kg. Then, a total of 576 growing barrows weighing approximately 25 kg were blocked by body weight and housed in 72 pens with 8 pigs per pen. Each group of pigs (*n* = 96 pigs) was fed one of the six experimental diets (Table 1), with 12 pens per diet according to a randomized completely block design. The experimental period was divided into 4 phases based on body weight according to NRC (2012) recommendations: phase 1 (25 to 50 kg), phase 2 (50 to 75 kg), phase 3 (75 to 100 kg), and phase 4 (100 to 135 kg), and lasted 17 weeks with 5 weeks for phase 1 and 4 weeks for each of the following 3 phases.

### 2.2. Experimental Diets

In each feeding phase, a total of six experimental diets were formulated. Three of the diets were without any supplemental multi-carbohydrase and phytase complex (MCPC) (Table 1 and Table 2), including a positive control diet (PC), negative control diet 1 (NC1), and negative control diet 2 (NC2). Another three diets (PC + MCPC, NC1 + MCPC and NC2 + MCPC) were with supplemental 100 g of MCPC per metric ton to the basal diets and supplied at least 1800 U of xylanase, 1244 U of beta-glucanase, 6600 U of a-arabinofuranosidase, and 1000 FTU of phytase (Rovabio, Advance Phy, Adisseo France SAS, France) per kg of diet. The PC diets, based on corn, soybean meal, wheat (fixed at 10%), wheat bran, and soy hulls, were formulated to be adequate but not excessive in net energy (NE), standard ileal digestible (SID) amino acids (AA), standard total-tract digestible (STTD) P, and total calcium (Ca), as recommended by the NRC (2012) (Table 2). The calculated NE and SID AA levels in NC1 diets were reduced to 97% of the PC diets, while STTD P and total Ca levels in NC1 diets were reduced by 0.080% and 0.071% units, respectively, compared with the PC diets. The calculated NE and SID AA levels in NC2 diets were reduced to 95% of the PC diets, while STTD P and total Ca levels in NC2 diets were reduced by 0.080% and 0.071% units, respectively, compared with the PC diets (the detail as shown in Supplemental Table S2). The analyzed values of the composition of the experimental diets (phase 1) are shown in Table 3. The database (NRC 2012) was used to calculate NE, SID of amino acids, and STTD of P. All experimental diets were fed in mash form.

### 2.3. Animal Management

Pens (6 m × 2.3 m) were regularly cleaned, disinfected, and dried. All pigs were vaccinated and dewormed according to the routine management and immunization procedures of the farm. Pigs were fed at 07:30, 14:00 and 20:00 ad libitum and had free access to water throughout the experiment period. The feed consumption of each pen was recorded weekly. At the end of each phase, the total body weight of each pen of pigs was recorded after removing the feed for 12 h to calculate the average weight. Net energy intake was determined by multiplying the total feed intake by the NE content of each diet and dividing it by the number of days in each phase. Net energy: gain ratio (NE:G) was determined by dividing the total NE intake by the weight gain in each phase. Room temperature was set to 22–25 °C.

Chromium trioxide (0.3% of diet) was added as an indigestible marker in each diet during the last two weeks of feeding phase 1 (25 to 50 kg BW). Fresh fecal samples were collected from each pen for four consecutive days after the pigs had ten days of adapting to the diets. Fecal samples were stored at −20 °C until analysis. In addition, at the end of phase 1, one pig from each pen was randomly selected, weighed, and slaughtered by penetrating captive bolt; then, ileal content was collected by gentle squeezing and stored at −20 °C until analysis. At the end of the experiment, one pig per pen with mean body weight, a total of 12 pigs per diet, were euthanized for carcass trait measurement.

### 2.4. Sample Preparation and Analysis

Fecal samples were dried in a fan-forced oven at 65 °C for 72 h, while ileal digesta samples were dried by vacuum freeze drying, then ground to pass a 1-mm sieve. Representative samples of experimental diets used in feeding phase 1 (25–50 kg BW), digesta and fecal samples were taken and analyzed in duplicate for proximate nutrients, Ca, total P, neutral detergent fiber (NDF), acid detergent fiber (ADF), and chromium [18]. Crude protein (CP) was analyzed by the Kjeldahl nitrogen method (AOAC method 988.05). Gross energy was determined by calorimeter (parr6400 CALORIMETER, Moline, IL, USA). Total P (AOAC method 965.05) and chromium (AOAC method 974.27) were determined by microplate spectrophotometer (Spectramax 190, Molecular Devices, Sunnyvale, CA, USA). Ca was analyzed by the potassium permanganate titration method (AOAC method 927.02). NDF and ADF (AOAC method 973.18) were determined by an Automatic fiber analyzer (ANKOM2000i, Ankom Technology, Macedon, NY, USA). The AA concentration of hydrolyzed protein was determined by ion-exchange chromatography with an automatic amino acid analyzer (LA8080 HITACHI, Tokyo, Japan).

The AID and ATTD of nutrients in the diet were calculated by the following formula:

$$\mathrm{AID}\;\mathrm{or}\;\mathrm{ATTD}\left(\%\right)=100-\frac{{\mathrm{Cr}}_{2}O_{3}\;\mathrm{in}\;\mathrm{feed}\left(\%\right)}{{\mathrm{Cr}}_{2}O_{3}\;\mathrm{in}\;\mathrm{feces}\;\mathrm{or}\;\mathrm{digesta}\left(\%\right)}\times \frac{\mathrm{Components}\;\mathrm{content}\;\mathrm{in}\;\mathrm{feces}\;\mathrm{or}\;\mathrm{digesta}\left(\%\right)}{\mathrm{Components}\;\mathrm{content}\;\mathrm{in}\;\mathrm{feed}\left(\%\right)}\times 100$$

The left tibia was excised from each euthanized pig for bone-breaking strength and ash, Ca, and P contents determination. The left tibia samples were de-fleshed and cleaned by scalpel blades, and the bone-breaking strength was determined by Bone Strength Tester (Wuhan Huatuo Measurement Technology Co., Ltd., Wuhan, China). The tibia samples were defatted by soaking in petroleum ether for 72 h to remove the fat before ashing. Then, the tibia samples were dried to a constant weight by a fan-forced oven at 105 °C. The dry, defatted tibias samples were ground and accurately weighed and ashed in a 550 °C muffle furnace for 12–16 h to determine ash, Ca, and P contents.

### 2.5. Statistical Analysis

The growth performance was analyzed using the PROC MIXED procedure of SAS (SAS 9.4, Inst. Inc., Cary, NC, USA) with a two-way factorial arrangement on randomized complete block design. The first factor consisted of the add MCPC and without MCPC. The second factor consisted of three levels of diet (PC, NC1, NC2). Pen is the experimental unit, while initial body weight is the covariance. The effect of dietary treatment was determined using specific contrasts. The data of ATTD, AID, Bone index and carcass traits were analyzed by the two-way ANOVA procedure. *p* < 0.05 was considered significant.

## 3. Results

### 3.1. Growth Performance

The effects of supplemental MCPC in diets differing in nutritional levels on the growth performance of growing–finishing pigs are presented in Table 4. NC2 had lower (*p* < 0.01 and *p* < 0.05, respectively) final BW and overall (25–130 kg) ADG than PC and NC1 diets. NC1 and NC2 diets had higher (*p* < 0.05) ADFI and feed-gain ratio than the PC diet during 25–100 kg and 25–130 kg periods, while NC2 also had a higher (*p* < 0.05) feed-gain ratio than the PC diet during 25–50 kg and 25–75 kg period. MCPC supplementation had a significant effect on BW at the end of phases 1 (*p* < 0.001), 2 (*p* < 0.05), and 4 (*p* < 0.05), on ADG (*p* < 0.001) during 25–50 kg periods, on NE:G (*p* < 0.05) during the 25–50 kg period, in addition to the ADFI (*p* < 0.05) and feed-gain ratio (*p* < 0.05) during 25–50 kg and 25–75 kg period. Specifically, MCPC supplementation resulted in growth rate, feed intake, and feed efficiency improvements by 0.57%, 2.55%, and 6%, respectively, during the 25–50 kg period. Furthermore, significant diet × MCPC interaction was observed on BW at the end of phase 4. No significant diet × MCPC interaction was observed in ADG, ADFI, and feed-gain ratio throughout the experimental period.

### 3.2. Bone Strength and Mineralization

**Figure 1:**
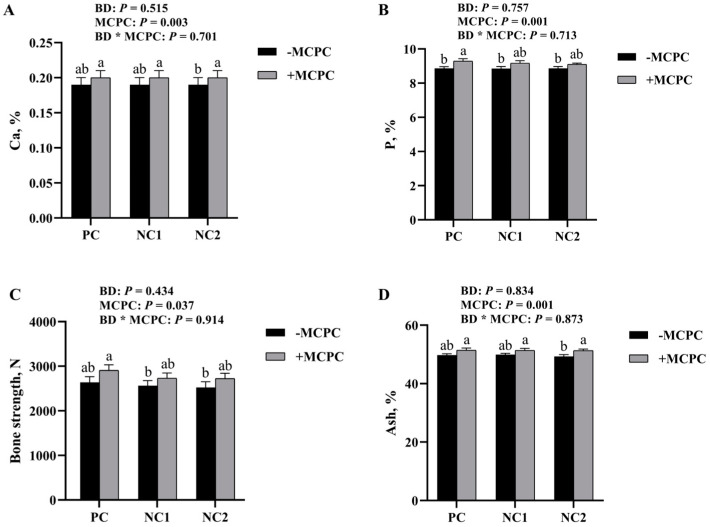
Effects of supplemental multi-enzyme (MCPC) in diets differing in nutritional levels on calcium (Ca), phosphorus (P), bone strength and Ash percentage in bone of growing–finishing pigs (25–50 kg). (**A**) the Ca percentage in bone of growing-finishing pigs; (**B**) the P percentage in bone of growing-finishing pigs; (**C**) the bone strength of growing-finishing pigs; (**D**) the Ash percentage in bone of growing-finishing pigs. PC = positive control; NC1 = negative control 1; NC2 = negative control 2; MCPC = multi-carbohydrase and phytase complex. a, b, c means with no common letters differ at *p* < 0.05. *n* = 12 pigs/treatment.

The bone strength (*p* = 0.430 and *p* = 0.910, respectively) and Ca (*p* = 0.510 and *p* = 0.700, respectively), P (*p* = 0.760 and *p* = 0.710, respectively), and ash (*p* = 0.830 and *p* = 0.870, respectively) percentage in bone were not significantly (*p* > 0.10) affected by the basal diet or the interaction of diet × MCPC (Figure 1). MCPC showed a significant (*p* < 0.05) positive effect on bone strength, Ca, P, and ash percentage in bone by +8.3%, +4.6%, +3.6% and +3.0%, respectively, in comparison to diets without MCPC.

### 3.3. Digestibility Coefficients

The effects of basal diet and MCPC supplementation on apparent ileal digestibility (AID) coefficients of AA in growing pigs (25–50 kg) are shown in Table 5. Diet showed a significant (*p* < 0.05) effect on AID coefficients of Lys, Met, Cys, Thr, Ile, Asp, Ser, Tyr, and Arg. Particularly, the AID coefficient of Met, Cys, Thr, Asp, Ser, Tyr, and Arg were lower (*p* < 0.05 or *p* < 0.01) in NC1 and NC2 than in the PC diet, whereas the AID coefficient of Lys and Ile were lower (*p* < 0.01 or *p* < 0.05, respectively) in NC1 than in PC diet. MCPC improved significantly (*p* < 0.05) the AID of Lys, Leu, Val, Phe, Gly, and Pro. The AID of AA was not significantly (*p* > 0.10) affected by the interaction of diet × MCPC (Table 5).

The type of basal diet showed a significant (*p* < 0.05) effect on the AID coefficient of GE and CP. Specifically, AID coefficients of GE decreased (*p* < 0.05) in NC2 and tended (*p* < 0.10) to decrease in NC1, while that of CP decreased (*p* < 0.05) in NC1 and tended (*p* < 0.10) to decrease in NC2 when compared with the PC diet (Table 6). No significant effect of the basal diet was found on AID of Ca and P (Supplemental Figure S1), while MCPC supplementation resulted in a significant (*p* < 0.05) improvement of the AID coefficient of Ca (+33.2%) and P (+115.7%).

The effects of the basal diet and MCPC supplementation on diets with different levels of nutrients on apparent total-tract digestibility (ATTD) coefficients of GE, CP, Ca, P, NDF and ADF of growing pigs (25–50 kg BW) are shown in Table 7. Basal diet significantly (*p* < 0.05) affected ATTD coefficients of ADF and tended to affect ATTD coefficients of GE (*p* = 0.08), Ca (*p* = 0.08) and NDF (*p* = 0.07). MCPC supplementation significantly (*p* < 0.05) improved ATTD coefficients of GE, CP, and P. Interestingly, ATTD coefficients of NDF and ADF were significantly (*p* < 0.05) affected by the interaction of diet × MCPC. Indeed, ATTD coefficients of NDF and ADF were improved (*p* < 0.05) in the PC diet, whereas no effect (*p* > 0.10) in either NC1 or NC2 was observed following MCPC supplementation (Supplemental Figure S2).

### 3.4. Carcass Traits

The results on carcass traits of pigs at slaughter age (around 130 kg live weight) are presented in Table 8. NC2 diet decreased (*p* < 0.05) carcass weight compared to PC and NC1 diets and increased (*p* < 0.05) lean meat percentage compared to the PC diet, whereas no difference was observed between the NC1 and PC diets. Given that these pigs were selected according to the mean final body weight of each treatment, the difference in carcass traits was mainly associated with the live weight, which was not different (*p* > 0.10) between PC-and NC1-fed pigs but significantly (*p* < 0.05) lower in NC2 than in PC-fed pigs. MCPC supplementation resulted in a significant (*p* < 0.05) improvement in carcass weight (+2.7%).

## 4. Discussion

There is growing interest in minimizing the cost of dietary formulation by using particular feed by-products supplemented with exogenous enzymes, targeting main antinutrients (phytic acid and NSP) in monogastric animals. Studies performed on poultry have suggested that the use of enzyme complexes containing various carbohydrase and phytase activities would allow for the reduction of dietary metabolizable energy and amino acid levels while maintaining optimum growth performance [16,19]. However, few study reports are available on the responses of growing–finishing pigs to the supplementation of enzyme complexes in diets with different nutritional levels. In the present study, the lower final body weight of pigs fed the NC2 diet than fed the NC1 and PC diets indicated the adverse effect of reduced nutrient levels on the growth rate of growing–finishing pigs, although they were fed ad-libitum. The higher feed intake of pigs fed NC1 and NC2 diets than fed PC diets during every experimental period suggested that pigs might meet their energy needs by adjusting feed intake. These things considered, the current study found that pigs fed NCI and NC2 diets exhibited lower nutrient digestibility. In totality, the reasons may further explain the higher feed–gain ratio of pigs fed NC1 and NC2 diets than fed PC diets during every experimental period. In addition, we found that reduced nutrient levels did not affect NE:G. The marked increase in both growth rate and feed efficiency observed in the 25–50 kg period might be explained by the fact that young pigs are more sensitive to the antinutritional effect of NSP or phytate due to their relatively immature digestive physiology. Indeed, improved performance following enzyme-complex addition in weaned pigs [20,21], whereas inconsistent results have been obtained in growing–finishing pigs following dietary supplementation with enzyme complex [22]. Otherwise, in this study, we found that NE:G decreased when MCPC was added at the 25–50 kg stage. MCPC is beneficial to pigs who consume less energy per unit of weight gain.

NSP are the main components of the cell walls of plant feeds, including corn, soya, and cereal grains such as wheat and barley. The antinutritional effect of NSP is, on the one hand, ascribed to the viscosity of soluble NSP such as arabinoxylans and, on the other hand, associated with the physical encapsulation of NSP, thereby preventing the release of intracellular nutrients, including proteins and starch. In addition, phytate, also known as an antinutrient in monogastric animals, was increased by 11% to 21%, respectively, in NC1 and NC2 diets in comparison with the PC diet. The significant reduction in AID of GE, CP, and AA observed in the present study in animals fed NC1 and NC2 diets probably reflect the antinutritional effects of arabinoxylans and phytate on the digestive process. Indeed, it has been documented that arabinoxylans decreased the digestibility of energy and amino acids in pigs [23], while phytate affected the digestibility of minerals and amino acids and the bioavailability of energy in both poultry and pigs [24,25]. In the present study, the multi-enzyme supplementation showed a potent role in improving AID of AA, particularly Lys, Leu, Val, Phe, Gly, and Pro, and ATTD of GE and CP. The increased digestibility of energy and AA partly explained the improved growth performance of pigs, which agreed well with the previous studies in weaned piglets [20,21] and growing–finishing pigs [12,13,22]. Similarly, studies in broilers also reported that optimal growth performance was obtained by using enzyme complexes containing carbohydrase and phytase activities in diets at low metabolizable energy and AA levels [17,19,23]. The improved energy and AA digestibility could be explained by the fact that xylanase, beta-glucanase, and arabinofuranosidase could destroy plant cell wall structure and thus facilitate the release and subsequent digestion of intracellular proteins and starch by endogenous enzymes [26]. In addition, a previous study has shown that exogenous enzymes reduce the loss of endogenous nitrogen, which may partly explain the higher protein/amino acids digestibility of pigs fed MCPC supplemental diets [27]. In addition, the growth rate of weaned pigs was not affected by the single addition of xylanase [24]. These results might suggest the synergistic effect of beta-glucanase and/or debranching enzyme (arabinofuranosidase) with xylanase in facilitating the release of intracellular nutrients. Otherwise, according to diet formulation and analysis, we found that measured ileal digestibility values are lower and more different among treatments than expected. This may be related to the difference in feed materials, sample collection and index measurement, and significant diet × MCPC interaction was observed on ADF and NDF in this study. The dietary composition had different effects on the effect of exogenous enzymes [28].

Phytate is another antinutritional factor commonly existing in feedstuffs. Phytate, as the primary storage form of P, accounts for 65–80% of the total P in cereal grains [1], and more than 60% of P in corn is also present in the form of phytate [2]. Phytate not only hinders the digestion of P but also binds proteins and other minerals such as Ca and Zn, thereby reducing nutrient digestibility and growth performance [7,8]. In the present study, the designed STTD P and total Ca levels in both NC1 and NC2 diets were reduced similarly by 0.080% and 0.071% units, respectively, compared with the PC diets. However, diets showed no significant effect on bone strength, the content of Ca, P, and ash in bone, and apparent ileal or total-tract digestibility of Ca and P. This might be explained by two aspects. Firstly, the designed difference in Ca and digestible P levels between diets may not be larger enough to cause a significant difference in the digestibility of Ca and P and their retention in bones. Secondly, the difference in the actual content of Ca and digestible P between diets, as reflected by the analyzed values, was not as large as that indicated by the designed levels. In support of this, among the three basal diets without enzyme supplementation, numerically higher AID coefficients of Ca and P were observed in the NC1 and NC2 diets compared with the PC diet (Supplemental Figure S2). Moreover, enzyme supplementation did show a significant effect on improving apparent ileal and/or total-tract digestibility coefficients of Ca and P and their retention in bones. In agreement with our results, many studies have shown that phytase can effectively increase Ca and P digestion and utilization [11,29,30] and thus make it feasible to lower the inclusion levels of Ca and P in diets [31,32]. Given that multi-carbohydrase and phytase were supplemented in our experimental diets in combination, it is possible that the enhanced phytate degradation has also contributed to the improvement of energy and AA digestibility. Indeed, a cocktail of carbohydrases and phytases was reported to significantly increase ATTD of gross energy, P, and Ca in corn or wheat-based diets for weanling and growing–finishing pigs [12]. Such improvement, especially in P and nitrogen digestibility, could be an interesting approach to reducing N and P excretion, thereby supporting sustainable swine production.

Furthermore, in the present study, compared with the PC diet, the lower carcass weight and higher lean meat percentage were observed in NC2 rather than in the NC1 diet. Considering that NC1 and NC2 diets had the same magnitude reduction of Ca and digestible P compared with the PC diet, the NE and digestible amino acid levels were different between NC1 and NC2. Shelton et al. studied a tendency (*p* = 0.100) towards a decrease in final body weight, hot carcass weight, and lean carcass weight in pigs fed the low Ca and P diet, whereas these responses could be reversed by dietary supplementation with phytase. Thus, the difference in the magnitude of Ca (0.080% vs. 0.100%) and P (0.071% vs. 0.100%) reduction might explain the discrepancy in the responses of carcass traits between present and previous studies [33]. Furthermore, Kerr et al. found that dietary crude protein and NE failed to interact on any carcass variable measured [34]. In this study, the difference between the average final BW and average slaughter BW (the average final BW and the average slaughter were 1.9 kg and 4.7 kg difference between extreme values PC vs. NC2, respectively. Thus, the effect of diet on carcass weight may be related to slaughter selection.

## 5. Conclusions

The MCPC (xylanase, beta-glucanase, arabinofuranosidase, and phytase) had a potent role in improving energy, AA, Ca, and P digestibility in growing pigs regardless of dietary nutrient levels, thus increasing the deposition of Ca and P in bone and improving the growth rate and feed efficiency of growing pigs.

## Figures and Tables

**Table 1:** Ingredients of the basal diets including positive control (PC), negative controls (NC) 1 and 2 for each phase (%, as-fed basis).

Item	Phase 1 (25–50 kg BW)			Phase 2 (50–75 kg BW)			Phase 3 (75–100 kg BW)			Phase 4 (100–135 kg BW)		
	PC	NC1	NC2	PC	NC1	NC2	PC	NC1	NC2	PC	NC1	NC2
Corn	58.91	56.41	52.10	63.75	58.67	54.83	67.73	62.22	58.58	68.89	63.62	60.02
Soybean meal (CP 46%)	14.10	12.43	11.61	9.95	9.05	7.90	6.32	5.70	4.78	5.50	4.20	3.50
Wheat bran	10.00	15.00	20.00	10.00	15.30	20.20	10.00	15.65	20.40	10.00	16.00	20.55
Wheat (CP 11%)	10.00	10.00	10.00	10.00	10.00	10.00	10.00	10.00	10.00	10.00	10.00	10.00
Soy hulls	0.90	1.20	1.32	1.26	2.60	2.70	1.70	2.82	2.72	1.98	3.14	3.00
Soybean oil	2.57	1.90	1.97	1.93	1.79	1.81	1.45	1.35	1.30	1.30	1.20	1.12
Limestone	1.15	1.08	1.07	1.04	0.96	0.95	0.92	0.83	0.83	0.81	0.73	0.72
Monocalcium phosphate	0.96	0.59	0.58	0.80	0.43	0.43	0.70	0.32	0.32	0.57	0.20	0.19
L-Lysine HCl (98%)	0.53	0.52	0.51	0.49	0.46	0.46	0.45	0.42	0.41	0.32	0.31	0.30
DL-Methionine (99%)	0.11	0.10	0.09	0.08	0.06	0.06	0.05	0.03	0.03	0.01	0.01	0.01
L-Threonine	0.18	0.17	0.17	0.16	0.15	0.15	0.15	0.13	0.13	0.10	0.09	0.09
L-Tryptophane	0.03	0.03	0.02	0.03	0.03	0.02	0.03	0.02	0.02	0.01	0.01	0.01
L-Valine	0.08	0.07	0.05	0.05	0.03	0.03	0.04	0.03	0.02	0.04	0.03	0.02
NaCl	0.23	0.23	0.23	0.23	0.23	0.23	0.24	0.24	0.24	0.24	0.24	0.24
Premix ^1^	0.27	0.27	0.27	0.23	0.23	0.23	0.23	0.23	0.23	0.23	0.23	0.23
Total	100.00	100.00	100.00	100.00	100.00	100.00	100.00	100.00	100.00	100.00	100.00	100.00

^1^ Provided per kg of diet for 25–50 kg and >50 kg phases: vitamin premix 300 mg, 250 mg (vitamin A, 13,500, 11,250 IU; vitamin D_3_, 2550, 2125 IU; vitamin E, 25, 21 mg; menadione, 3, 2.5 mg; thiamine, 2.4, 2 mg; riboflavin, 6, 5 mg; niacin, 30, 25 mg; d-pantothenic acid, 15, 12.5 mg; vitamin B_6_, 2.4, 2 mg; vitamin B_12_, 30, 25 μg; d-biotin, 150, 125 μg; folic acid, 1.5, 1.25 mg), choline 400, 300 mg; copper, 8, 6 mg; iron, 90, 60 mg; manganese, 4 mg; zinc, 90, 75 mg; iodine, 0.28 mg; selenium, 0.3 mg; flavor, 200 mg; antioxidant, 100 mg; anti-mold, 500 mg; Oregano oil, 200 mg.

**Table 2:** Nutrient characteristics of the basal diets including positive control (PC) and negative control (NC) 1 and 2 (Calculated values).

Item	Phase 1 (25 to 50 kg BW)			Phase 2 (50 to 75 kg BW)			Phase 3 (75 to 100 kg BW)			Phase 4 (100 to 135 kg BW)		
	PC	NC1	NC2	PC	NC1	NC2	PC	NC1	NC2	PC	NC1	NC2
Dry Matter, %	86.47	86.74	86.79	86.46	86.83	86.88	86.42	86.81	86.84	86.50	86.88	86.91
Crude protein, %	14.56	14.40	14.41	13.04	13.12	13.03	11.71	11.90	11.84	11.23	11.24	11.27
Crude fat, %	5.50	4.89	4.96	5.05	4.89	4.89	4.64	4.51	4.48	4.55	4.44	4.36
Crude fiber, %	3.62	4.00	4.36	3.57	4.35	4.68	3.53	4.27	4.57	3.63	4.37	4.64
Ash, %	4.33	4.13	4.27	3.94	3.81	3.93	3.58	3.47	3.58	3.36	3.23	3.34
Net energy, kcal/kg	2475	2401	2351	2475	2401	2351	2475	2401	2351	2475	2401	2351
Digestible lysine, %	0.98	0.95	0.93	0.85	0.83	0.81	0.73	0.71	0.69	0.61	0.59	0.58
Digestible methionine, %	0.32	0.31	0.30	0.27	0.25	0.25	0.22	0.21	0.20	0.18	0.18	0.18
Digestible methionine + cysteine, %	0.55	0.53	0.52	0.48	0.47	0.46	0.42	0.41	0.40	0.38	0.37	0.37
Digestible threonine, %	0.59	0.57	0.56	0.52	0.50	0.49	0.46	0.45	0.44	0.40	0.39	0.38
Digestible tryptophan, %	0.17	0.17	0.16	0.15	0.15	0.14	0.13	0.13	0.12	0.11	0.11	0.11
Digestible arginine, %	0.78	0.76	0.76	0.67	0.67	0.66	0.58	0.59	0.58	0.55	0.55	0.54
Digestible histidine, %	0.34	0.33	0.33	0.31	0.31	0.30	0.28	0.28	0.28	0.27	0.27	0.27
Digestible isoleucine, %	0.51	0.49	0.48	0.45	0.44	0.43	0.40	0.39	0.38	0.38	0.37	0.36
Digestible leucine, %	1.17	1.13	1.10	1.10	1.06	1.02	1.04	1.01	0.97	1.02	0.98	0.95
Digestible phenylalanine, %	0.62	0.60	0.59	0.56	0.55	0.53	0.50	0.49	0.48	0.49	0.47	0.46
Digestible valine, %	0.64	0.62	0.61	0.55	0.53	0.52	0.48	0.48	0.47	0.47	0.46	0.45
Calcium, %	0.66	0.59	0.59	0.59	0.52	0.52	0.52	0.45	0.45	0.46	0.39	0.39
Total phosphorus, %	0.58	0.53	0.56	0.53	0.48	0.51	0.50	0.45	0.48	0.47	0.42	0.45
STTD-P, %	0.31	0.23	0.23	0.27	0.19	0.19	0.24	0.16	0.16	0.21	0.13	0.13
Sodium, %	0.10	0.10	0.10	0.10	0.10	0.10	0.10	0.10	0.10	0.10	0.10	0.10

The database (NRC 2012) was used to calculate NE, SID of amino acids, and STTD of P. Another three diets were supplemented with 100 g/t MCPC on the basal diets, and the MCPC provided at least 1800 U of xylanase, 1244 U of beta-glucanase, 6600 U of a-arabinofuranosidase, and 1000 FTU of phytase (Rovabio, Advance Phy, Adisseo France SAS, Antony, France) per kg of diet.

**Table 3:** Nutrient characteristics of the basal diets including positive control (PC) and negative control (NC) 1 and 2 (Analyzed values).

Item	Phase 1 (25–50 kg BW)		
	PC	NC1	NC2
Gross energy, kcal/kg	3916	3908	3877
Crude protein, %	14.75	14.80	14.73
Neutral detergent fiber (NDF), %	15.89	16.17	16.38
Acid detergent fiber (ADF), %	12.90	12.13	11.94
Lysine, %	1.15	1.10	1.10
Methionine, %	0.40	0.32	0.30
Cysteine, %	0.29	0.26	0.29
Threonine, %	0.76	0.71	0.71
Isoleucine, %	0.58	0.55	0.54
Leucine, %	1.37	1.33	1.31
Phenylalanine, %	0.82	0.83	0.86
Histidine, %	0.42	0.42	0.43
Aspartic acid, %	1.37	1.27	1.25
Serine, %	0.77	0.71	0.72
Glutamic acid, %	2.87	2.83	2.80
Glycine, %	0.60	0.61	0.61
Tyrosine, %	0.44	0.42	0.42
Arginine, %	0.80	0.82	0.78
Proline, %	1.01	0.99	1.00
Alanine, %	0.78	0.76	0.74
Calcium, %	0.80	0.76	0.77
Total Phosphorus, %	0.55	0.53	0.49

**Table 4:** Effects of supplemental multi-enzyme (MCPC) in diets differing in nutritional levels on growth performance of growing–finishing pigs.

Item	Basal Diet (BD)			Enzyme (MCPC)		SEM	*p*-Value		
	PC	NC1	NC2	−MCPC	+MCPC		BD	MCPC	BD × MCPC
BW, kg									
Initial	24.70	24.69	24.74	24.71	24.71	0.51	0.193	0.872	0.607
At the end of phase 1	50.16	49.52	49.97	49.28 ^b^	50.48 ^a^	0.61	0.228	<0.001	0.880
At the end of phase 2	76.39	75.99	76.22	75.62 ^b^	76.78 ^a^	0.70	0.817	0.032	0.822
At the end of phase 3	103.81	104.02	102.53	102.87	104.04	0.77	0.248	0.139	0.110
At the end of phase 4	125.48 ^a^	126.89 ^a^	123.11 ^b^	124.23 ^b^	126.09 ^a^	0.70	<0.001	0.016	0.020
NE:G									
25–50 kg	5511.06	5522.42	5377.51	5537.05 ^b^	5403.62 ^a^	38.64	0.136	0.044	0.900
25–75 kg	5135.75	5127.24	5036.54	5109.78	5089.91	27.70	0.062	0.594	0.598
25–100kg	6739.79	6771.45	6671.80	6748.25	6707.11	31.90	0.203	0.374	0.277
25–130 kg	7211.04	7292.01	7217.21	7271.16	7209.01	33.23	0.282	0.180	0.200
Average daily gain (ADG), kg/d									
25–50 kg	0.727	0.709	0.721	0.702 ^b^	0.706 ^a^	0.01	0.234	<0.001	0.890
25–75 kg	0.979	0.970	0.971	0.961 ^b^	0.985 ^a^	0.01	0.737	0.019	0.974
25–100kg	0.871	0.872	0.854	0.859	0.872	0.01	0.178	0.140	0.170
25–130 kg	0.868 ^a^	0.874 ^a^	0.857 ^b^	0.870	0.862	0.01	0.005	0.060	0.070
Average daily feed intake (ADFI), kg/d									
25–50 kg	1.621	1.626	1.649	1.611 ^b^	1.652 ^a^	0.01	0.212	0.004	0.961
25–75 kg	2.032	2.072	2.081	2.040 ^b^	2.083 ^a^	0.02	0.148	0.042	0.748
25–100kg	2.373 ^a^	2.457 ^b^	2.423 ^ab^	2.401	2.428	0.02	0.036	0.429	0.599
25–130 kg	2.529 ^a^	2.655 ^b^	2.600 ^bc^	2.586	2.603	0.02	<0.001	0.458	0.146
Feed:gain ratio, kg/kg									
25–50 kg	2.227	2.300	2.288	2.299 ^a^	2.437 ^b^	0.02	0.066	0.043	0.884
25–75 kg	2.438 ^a^	2.515 ^b^	2.515 ^bc^	2.497	2.481	0.01	<0.001	0.384	0.842
25–100kg	2.679 ^a^	2.773 ^b^	2.799 ^bc^	2.756	2.745	0.02	<0.001	0.631	0.147
25–130 kg	2.914 ^a^	3.037 ^b^	3.070 ^bc^	3.019	2.994	0.02	<0.010	0.184	0.209

^a,b,c^ Means within a row with no common letters differ at *p* < 0.05 or *p* < 0.01. *n* = 12 pigs/treatment.

**Table 5:** Effects of supplemental multi-enzyme (MCPC) in diets differing in nutritional levels on apparent ileal digestibility (AID) coefficients of amino acids of growing–finishing pigs (25–50 kg).

Item	Basal Diet (BD)			Enzyme (MCPC)		SEM	*p*-Value		
	PC	NC1	NC2	−MCPC	+MCPC		BD	MCPC	BD × MCPC
Lysine	0.770 ^a^	0.722 ^b,^**	0.745 ^ab^	0.727 ^b^	0.764 ^a^	0.017	0.025	0.010	0.387
Methionine	0.827 ^a^	0.757 ^b,^**	0.764 ^b,^*	0.778	0.787	0.024	0.010	0.629	0.939
Cysteine	0.619 ^a^	0.500 ^c,^**	0.583 ^bc,^*	0.546	0.589	0.033	0.003	0.121	0.036
Threonine	0.655 ^a^	0.563 ^b,^**	0.586 ^b,^*	0.587	0.616	0.026	0.002	0.162	0.486
Isoleucine	0.665 ^a^	0.575 ^b,^*	0.617 ^ab^	0.602	0.636	0.026	0.005	0.120	0.639
Leucine	0.727	0.685	0.713	0.692 ^b^	0.724 ^a^	0.019	0.090	0.045	0.572
Valine	0.686	0.643	0.677	0.650 ^b^	0.687 ^a^	0.020	0.089	0.028	0.337
Phenylalanine	0.712	0.702	0.743	0.698 ^b^	0.740 ^a^	0.018	0.072	0.007	0.189
Histidine	0.659	0.594	0.640	0.598	0.631	0.038	0.195	0.988	0.362
Aspartic acid	0.642 ^a^	0.542 ^b,^**	0.566 ^b,^**	0.565	0.602	0.028	0.002	0.119	0.406
Serine	0.664 ^a^	0.563 ^b,^**	0.590 ^b,^**	0.589	0.622	0.026	0.001	0.135	0.416
Glutamic acid	0.732	0.682	0.716	0.697	0.724	0.023	0.098	0.159	0.314
Glycine	0.365	0.216	0.257	0.212 ^b^	0.347 ^a^	0.079	0.167	0.043	0.132
Tyrosine	0.685 ^a^	0.585 ^b,^**	0.581 ^b,^**	0.594	0.640	0.030	0.001	0.063	0.714
Arginine	0.742 ^a^	0.652 ^b,^*	0.643 ^b,^*	0.674	0.683	0.023	<0.001	0.659	0.964
Proline	0.700	0.692	0.684	0.665^b^	0.718^a^	0.029	0.863	0.034	0.455
Alanine	0.626	0.564	0.603	0.578	0.625	0.028	0.097	0.099	0.337

PC = positive control; NC1 = negative control 1; NC2 = negative control 2; MCPC = multi-carbohydrase and phytase complex. ^a,b,c^ means within a row with no common letters differ at *p* < 0.05 or *p* < 0.01. * *p* < 0.05 and ** *p* < 0.01 compared with PC. *n* = 8 pigs/treatment.

**Table 6:** Effects of supplemental multi-enzyme (MCPC) in diets differing in nutritional levels on apparent ileal digestibility (AID) coefficients of gross energy (GE), crude protein (CP), dry matter (DM), calcium (Ca), and phosphorus (P) of growing pigs (25–50 kg).

Item	Basal Diet (BD)			Enzyme (MCPC)		SEM	*p*-Value		
	PC	NC1	NC2	−MCPC	+MCPC		BD	MCPC	BD × MCPC
GE	0.637 ^a^	0.599 ^ab,#^	0.588 ^b,^*	0.602	0.614	0.046	0.032	0.432	0.593
CP	0.592 ^a^	0.524 ^b,^**	0.546 ^ab,#^	0.538	0.570	0.060	0.029	0.118	0.893
DM	0.955	0.946	0.946	0.949	0.949	0.011	0.054	0.989	0.269
Ca	0.389	0.417	0.480	0.368 ^b^	0.490 ^a^	0.149	0.327	0.020	0.536
P	0.305	0.251	0.259	0.172 ^b^	0.371 ^a^	0.172	0.707	0.002	0.381

PC = positive control; NC1 = negative control 1; NC2 = negative control 2; MCPC = multi-carbohydrase and phytase complex. ^a,b^ means within a row with no common letters differ at *p* < 0.05 or *p* < 0.01. ^#^ *p* < 0.10, * *p* < 0.05, and ** *p* < 0.01 compared with PC. *n* = 12 pigs/treatment.

**Table 7:** Effects of supplemental multi-enzyme (MCPC) in diets differing in nutritional levels on apparent total-tract digestibility (ATTD) coefficients of gross energy (GE), crude protein (CP), Ca, P, neutral detergent fiber (NDF) and acid detergent fiber (ADF) of growing–finishing pigs (25–50 kg).

Item	PC		NC1		NC2		SEM	Basal Diet (BD)			Enzyme (MCPC)		*p*-Value		
	−MCPC	+MCPC	−MCPC	+MCPC	−MCPC	+MCPC		PC	NC1	NC2	−MCPC	+MCPC	BD	MCPC	BD × MCPC
GE	0.751 ^bc^	0.784 ^a^	0.771 ^ac^	0.778 ^ac^	0.739 ^b^	0.760 ^ab^	0.028	0.767	0.775	0.749	0.754 ^b^	0.774 ^a^	0.077	0.03	0.53
CP	0.632 ^b^	0.684 ^ab^	0.679 ^ab^	0.691 ^a^	0.633 ^b^	0.678 ^ab^	0.047	0.658	0.685	0.656	0.648 ^b^	0.685 ^a^	0.244	0.023	0.568
Ca	0.308 ^b^	0.408 ^ab^	0.415 ^ab^	0.435 ^ab^	0.441 ^ab^	0.500 ^a^	0.122	0.358	0.425	0.471	0.388	0.448	0.081	0.149	0.726
P	0.181 ^c^	0.457 ^a^	0.313 ^b^	0.416 ^ab^	0.178 ^c^	0.400 ^ab^	0.036	0.319	0.364	0.289	0.224 ^b^	0.425 ^a^	0.125	<0.001	0.058
NDF	0.338 ^bc^	0.477 ^a^	0.418 ^ab^	0.350 ^bc^	0.326 ^c^	0.340 ^bc^	0.082	0.408	0.384	0.333	0.361	0.389	0.072	0.297	0.009
ADF	0.520 ^bc^	0.625 ^a^	0.555 ^ab^	0.450 ^bc^	0.477 ^c^	0.540 ^bc^	0.064	0.573 ^a^	0.527 ^ab,#^	0.509 ^b,^*	0.517	0.555	0.042	0.078	0.008

PC = positive control; NC1 = negative control 1; NC2 = negative control 2; MCPC = multi-carbohydrase and phytase complex. ^a,b,c^ means within a row with no common letters differ at *p* < 0.05 or *p* < 0.01. ^#^ *p* < 0.10 and * *p* < 0.05 compared with PC. *n* = 12 pigs/treatment.

**Table 8:** Effects of supplemental multi-enzyme (MCPC) in diets differing in nutritional levels on carcass traits of pigs at slaughter age.

Items	Basal Diet (BD)			Enzyme (MCPC)		SEM	*p*-Value		
	PC	NC1	NC2	−MCPC	+MCPC		BD	MCPC	BD × MCPC
Live weight, kg	128.5 ^a^	128.2 ^a^	123.8 ^b,^**	125.5	128.2	1.760	0.015	0.070	0.830
Carcass weight, kg	96.0 ^a^	96.0 ^a^	927 ^b,^*	93.6 ^b^	96.2 ^a^	1.450	0.033	0.037	0.821
Carcass yield, %	74.8	74.9	74.9	74.6	75.0	0.003	0.817	0.126	0.517
Carcass length, cm	86.9	86.2	85.8	85.7	86.9	1.002	0.559	0.160	0.299
Backfat thickness, mm	26.5	27.1	25.3	26.4	26.2	1.396	0.449	0.825	0.976
Lean meat percentage, %	52.7 ^b^	54.8 ^ab^	55.6 ^a,^*	54.5	54.2	1.138	0.041	0.719	0.162
LM area, mm^2^	5188	4939	4858	5007	4983	268.9	0.445	0.912	0.798

PC = positive control; NC1 = negative control 1; NC2 = negative control 2; MCPC = multi-carbohydrase and phytase complex. ^a,b^ means within a row with no common letters differ at *p* < 0.05 or *p* < 0.01. * *p* < 0.05 and ** *p* < 0.01 compared with PC. *n* = 12 pigs/treatment.

## Data Availability

None of the data were deposited in an official repository. Data that support the present study are available upon request.

## Supplementary Materials

The following supporting information can be downloaded at: https://www.mdpi.com/article/10.3390/ani13091557/s1, Table S1: Ingredients and compositions of diets for 7–11 kg and 11–25 kg piglets; Table S2: The diets of descriptions. Figure S1: Effects of supplemental multi-enzyme (MCPC) in diets differing in nutritional levels on apparent ileal digestibility (AID) coefficients of Ca and P of growing–finishing pigs; Figure S2: Effects of supplemental multi-enzyme (MCPC) in diets differing in nutritional levels on apparent total-tract digestibility (ATTD) coefficients of neutral detergent fiber (NDF) and acid detergent fiber (ADF) of growing–finishing pigs.
