# Supplementary material for: Supplementation of a Multi-Carbohydrase and Phytase Complex in Diets Regardless of Nutritional Levels, Improved Nutrients Digestibility, Growth Performance, and Bone Mineralization of Growing–Finishing Pigs
Source: Animals (Basel). 2023 May 6;13(9):1557. doi: 10.3390/ani13091557 (PMC10177175; doi:10.3390/ani13091557)
Supplement: Supplementary file 1 [file animals-13-01557-s001.zip › animals-2245019-supplementary.pdf]

Supplemental Table S1. Ingredients and compositions of diets for 7-11 kg and 11-25 kg piglets.

Ingredients, %	7-11 kg	11-25 kg	Composition	7-11 kg	11-25 kg
Corn	40.423	47.091	Digestible energy, Kcal/kg	3557	3479
Whey powder	25.000	20.000	Metabolizable energy, Kcal/kg	3393	3336
Soybean meal, dehulled, extruded	16.200	19.000	Net engery, Kcal/kg	2448	2412
Fish meal	5.000	2.500	Crude protein, %	20.990	18.250
Porcine plasma	5.000	2.500	Ether extract, %	4.170	3.500
Heat-treated soybeans	5.000		Ash, %	3.070	2.680
Wheat bran	0.900	5.400	Crude fibre, %	1.730	2.310
Soybean hull	0.300	0.900	Digestible lysine, %	1.350	1.230
Lysine (78.8%)	0.214	0.391	Digestible methionine %	0.460	0.410
DL-methionine	0.120	0.141	Digestible methionine+cysteine, %	0.740	0.680
Threonine	0.019	0.100	Digestible threonine, %	0.790	0.730
Valine		0.083	Digestible tryptophan, %	0.240	0.200
Monocalcium phosphate	0.144	0.254	Digestible isoleucine, %	0.750	0.670
Limestone	0.715	0.810	Digestible valine, %	0.860	0.780
Sodium chloride	0.350	0.300	Digestible arginine, %	1.230	1.100
Sodium sulfate, decahydrate		0.100	Digestible histidine, %	0.490	0.430
Mineral premix[1]	0.090	0.090	Digestible leucine, %	1.570	1.390
Vitamin premix[2]	0.035	0.030	Digestible phenylalanine, %	0.880	0.770
Choline chloride (50%)	0.100	0.100	Digestible phenylalanine+tyrosine, %	1.450	1.250
Ethoxyquin (30%)	0.030	0.030	Calcium, %	0.800	0.700
Anti-mould additive	0.050	0.050	Total phosphorus, %	0.650	0.600
Flavour	0.050	0.050	Standard total tract digestible-phosphorus %	0.460	0.390

ZnO	0.200	
Antibiotic growth promoters[3]	0.060	0.060
Total	100.000	100.000

[1]Provided per kg of diet: copper, 200, 100 mg; iron, 100, 100 mg; manganese, 4, 3 mg; zinc, 100, 80 mg; iodine, 0.14, 0.14 mg; selenium, 0.30, 0.25mg, for 7-11 kg and 11-25 kg phases, respectively.

[2]Provided per kg of diet: vitamin A, 11375, 9750 IU; vitamin D_3, 3500, 3000 IU; vitamin E, 28, 24 mg; menadione, 3.5, 3 mg; thiamine, 3.5, 3 mg; riboflavin, 8.75, 7.5 mg; niacin, 35, 30 mg; d-panthothenic acid, 17.5, 15 mg; vitamin B6, 4.2, 3.6 mg; vitamin B12, 42, 36 μg; d-biotin, 175, 150 μg; folic acid, 1.75, 1.5 mg, for 7-11 kg and 11-25 kg phases, respectively.

[3]Provided per kg of diet: 12 mg of flavomycin and 75 mg of chlortetracycline via 12% of flavomycin premix and 15% of chlortetracycline premix, respectively.

Supplemental Table S2. The diets of descriptions.

Diet names	Descriptions
PC	Nutrient-adequate diet
NC1	PC reduced in NE, dig. AA, SID P, and Ca by -3%, -3%, and -0.080% unit, and -0.071% unit, respectively.
NC2	PC reduced in NE, dig. AA, SID P, and Ca by -5%, -5%, and -0.080% unit, and -0.071% unit, respectively.
PC + MCPC	PC supplemented with multi-enzyme at 100 g/ metric ton of feed
NC1 + MCPC	NC1 supplemented with multi-enzyme at 100 g/ metric ton of feed
NC2 + MCPC	NC2 supplemented with multi-enzyme at 100 g/ metric ton of feed

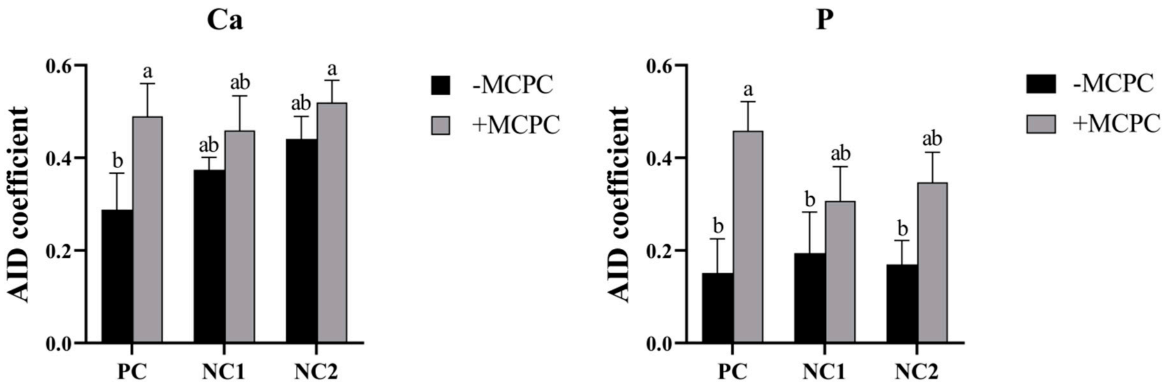

Supplemental Figure S1. Effects of supplemental multi-enzyme (MCPC) in diets differing in nutritional levels on apparent ileal digestibility (AID) coefficients of Ca and P of growing-finishing pigs.

PC = positive control; NC1 = negative control 1; NC2 = negative control 2; MCPC = multi-carbohydrase and phytase complex.

[a,b,c] Means with no common letters differ at $P < 0.05$.

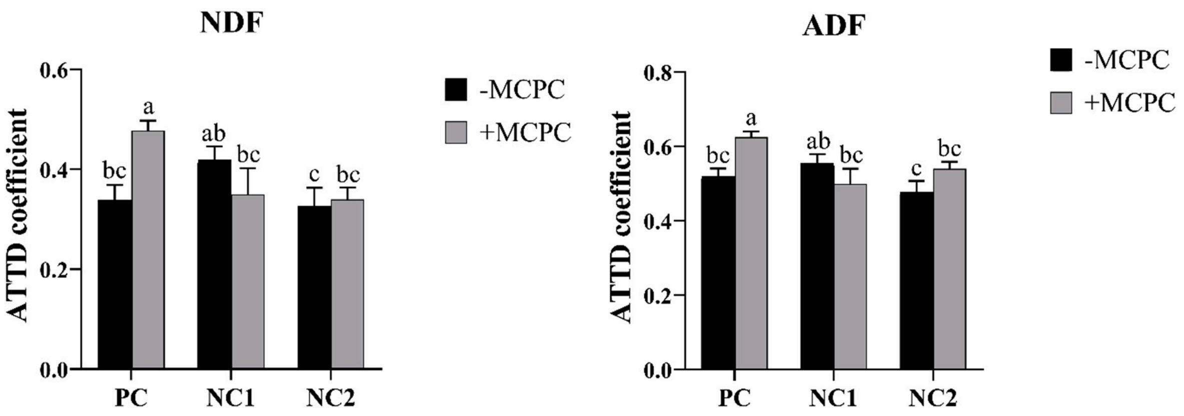

Supplemental Figure S2. Effects of supplemental multi-enzyme (MCPC) in diets differing in nutritional levels on apparent total-tract digestibility (ATTD) coefficients of neutral detergent fibre (NDF) and acid detergent fiber (ADF) of growing-finishing pigs.
PC = positive control; NC1 = negative control 1; NC2 = negative control 2; MCPC = multi-carbohydrase and phytase complex.

[a,b,c] Means with no common letters differ at $P < 0.05$.
